# Characterization of the Biosynthesis, Processing and Kinetic Mechanism of Action of the Enzyme Deficient in Mucopolysaccharidosis IIIC

**DOI:** 10.1371/journal.pone.0024951

**Published:** 2011-09-21

**Authors:** Xiaolian Fan, Ilona Tkachyova, Ankit Sinha, Brigitte Rigat, Don Mahuran

**Affiliations:** 1 Genetics and Genome Biology Program, The Hospital For Sick Children, Toronto, Canada; 2 Department of Laboratory Medicine and Pathobiology, University of Toronto, Toronto, Canada; Purdue University, United States of America

## Abstract

Heparin acetyl-CoA:alpha-glucosaminide N-acetyltransferase (N-acetyltransferase, EC 2.3.1.78) is an integral lysosomal membrane protein containing 11 transmembrane domains, encoded by the *HGSNAT* gene. Deficiencies of N-acetyltransferase lead to mucopolysaccharidosis IIIC. We demonstrate that contrary to a previous report, the N-acetyltransferase signal peptide is co-translationally cleaved and that this event is required for its intracellular transport to the lysosome. While we confirm that the N-acetyltransferase precursor polypeptide is processed in the lysosome into a small amino-terminal alpha- and a larger ß- chain, we further characterize this event by identifying the mature amino-terminus of each chain. We also demonstrate this processing step(s) is not, as previously reported, needed to produce a functional transferase, *i.e.*, the precursor is active. We next optimize the biochemical assay procedure so that it remains linear as N-acetyltransferase is purified or protein-extracts containing N-acetyltransferase are diluted, by the inclusion of negatively charged lipids. We then use this assay to demonstrate that the purified single N-acetyltransferase protein is both necessary and sufficient to express transferase activity, and that N-acetyltransferase functions as a monomer. Finally, the kinetic mechanism of action of purified N-acetyltransferase was evaluated and found to be a random sequential mechanism involving the formation of a ternary complex with its two substrates; i.e., N-acetyltransferase does not operate through a ping-pong mechanism as previously reported. We confirm this conclusion by demonstrating experimentally that no acetylated enzyme intermediate is formed during the reaction.

## Introduction

Mucopolysaccharidosis (MPS) IIIC (MIM: 252930), first described by Kresse et al. in 1978 [1], is an autosomal recessive disorder characterized by the lysosomal storage of heparin and heparan sulfate fragments containing terminal non-reducing alpha-glucosamine residues (alpha-GlcNH_2_). Clinically, MPS IIIC is similar to the other three forms of MPS III (A, B & D). MPS III has an occurrence of 1 in 66,000 births, with the IIIC form accounting for 1 in 1,407,000 (MPS Society of Australia, http://www.mpssociety.org.au/table_of_diseases.htm). Symptoms can vary in their severity, including severe central nervous system involvement, but only mild somatic manifestations [2]. Death usually occurs in the late teens. The defective enzyme is heparin acetyl-CoA: alpha-glucosaminide N-acetyltransferase, EC 2.3.1.78 (N-acetyltransferase) [3]. The gene encoding N-acetyltransferase, *HGSNAT*, was only identified in 2006 by us [4] and others [5]. Since then 61 mutations in the *HGSNAT* gene have been identified in MPS IIIC patients, 26 of which were missense [6], [7]. Understanding the biosynthesis and processing events leading to a functional N-acetyltransferase protein in lysosomes, as well as determining if the development of enzymatic activity is dependent on the oligomerization of the protein with itself and/or other gene products, as previously reported [8], [9], is essential for evaluating the potential effectiveness of therapeutic approaches for MPS IIIC, *e.g.*, small molecule enzyme enhancement therapy [10].

N-acetyltransferase is an integral membrane protein whose function is to acetylate the non-reducing, terminal alpha-GlcNH_2_ of intra-lysosomal fragments of heparin or heparan sulfate, converting it into a substrate for luminal alpha -N-acetyl glucosaminidase. However, it can also acetylate the ß-glucosamine residues of artificial substrates [11], which is the basis for the coupled (with ß-hexosaminidase) assay that is generally used for this transferase (see methods). Therefore, N-acetyltransferase catalyzes the only synthetic reaction known to occur in the lysosome. To accomplish this reaction, the enzyme uses a second cytosolic substrate, acetyl-coenzyme A (acetyl-CoA) [3]. Thus, the two substrates are separated by the lysosomal membrane. The mechanism by which N-acetyltransferase overcomes this spatial problem is controversial. The most accepted model is the ping-pong (double displacement) mechanism involving an initial acetylation reaction of the enzyme forming a covalently modified enzyme intermediate, E′, in the cytosol, followed by the transfer of the acetyl group to the intra-lysosomal heparin-a-glucosamine residue [8], [12], [13]. An alternative model proposes that the enzyme operates via a random-order ternary-complex mechanism where there is no preset order to the binding of the two substrates [14]; *i.e.*, there is no release of an initial product (CoA), nor production of a E′, prior to binding the second substrate (GlcNH_2_) and generation of the final product (N-acetylglucosamine).

**Figure 1:**
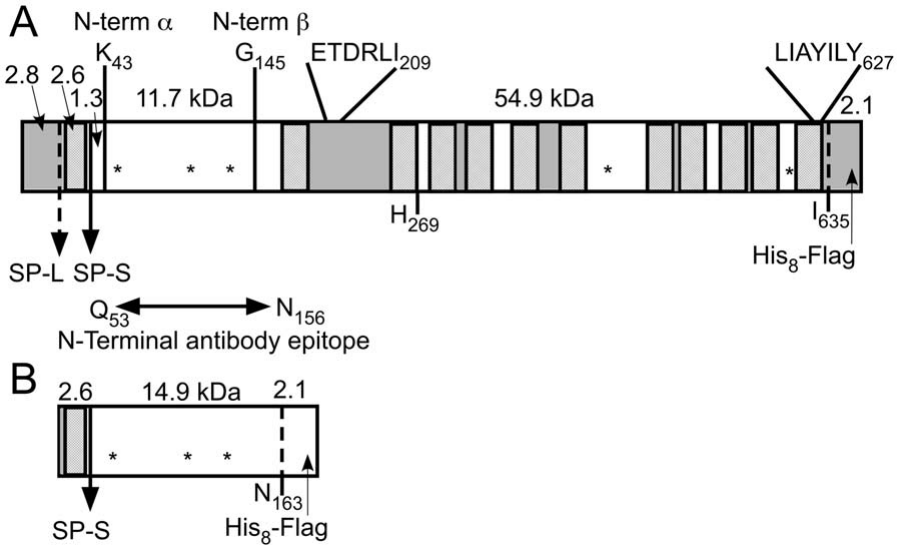
Scaled diagrams of the full length N-acetyltransferase and the small N-terminal loop fragment of the protein expressed by the cDNA constructs used in this study. Each construct also encodes a C-terminal His8-Flag tag. Predicted masses (kDa) are shown for each protein segment. TMDs are indicated by hatched rectangles, soluble loops that are predicted to reside in the ER/lysosomal lumen are shown as clear boxes and those predicted to reside in the cytosol as shaded boxes. Putative Asn-linked glycosylation sites (NXS/T) are shown as “*”. The location and ends of the peptide sequence used to generate the “N-terminal” antibody and utilized in this report, are shown between the two panels. (A) Full length N-acetyltransferase with both the long signal peptide (SP-L), found in the genomic sequence [13], and the short signal peptide (SP-S), used in this report and found in EST data bases are indicated. The positions and sequences of previously published intracellular transport signals [13] are shown, as are the N-termini, identified in this report, of the processed alpha- and ß- chains. (B) The N-terminal fragment containing the short signal peptide and the first luminal loop of N-acetyltransferase are depicted.

The deduced primary sequence of human N-acetyltransferase predicts a 635 amino acid protein containing a 30 amino acid amino-terminal (N-terminal) signal peptide, which, despite the presence of a computer-predicted signal peptide cleavage site between Gly30 and Arg31, has recently been reported to be non-cleavable [13], and 11 other transmembrane domains (TMDs). This degree of hydrophobicity partially explains why the enzyme has been so difficult to purify by conventional means (Fig. 1A). The number of TMDs suggests an orientation for the protein in which its small N-terminal tail (present only if the signal peptide is retained) is in the cytosol followed by the remaining 135 amino acid residues of the soluble N-terminal domain residing inside the ER, and ultimately the lysosome. The short 8 amino acid (Arg628-Ile635) carboxy-terminal (C-terminal) domain faces the cytosol (consistent with the survival of various C-terminal epitope tags after lysosomal compartmentalization [4], [5], [6], [13]). The predicted relative molecular mass (Mr) including the 30 amino acids of the signal peptide, is 70.5 kDa (72.6 kDa with our C-terminal His8Flag tag, Fig. 1A). There are also 5 consensus sites for Asn-linked glycosylation. Based on topology predictions we obtained through the TMHMM Server v.2.0, all of these Asn-X-Ser/Thr sites would be present in the lumen of the ER and thus available for glycosylation [4] (Fig. 1A). In general when SDS-PAGE is used to estimate the relative Mr of multi-TMD proteins, the results have been reported to vary as much as ±45% from those predicted from the deduced primary structures. This is believed to be caused by variations in the number of SDS molecules bound by the TMDs in comparison to the numbers bound by the similarly sized soluble proteins used as standards [15]. Additionally the presence of Asn-linked oligosaccharides can cause further deviations from the predicted Mr.

Whereas the deduced C-terminal half of N-acetyltransferase (exons 7–18) is highly conserved across species, including plants and bacteria; the N-terminus, extending back from the first TMD after the signal peptide, is only found in metazoans. Neither of these regions display any sequence homology with other previously identified functional domains, including those from proteins known to bind CoA or acetyl-CoA. Thus, the *HGSNAT*-encoded N-acetyltransferase represents the human member of a new family of enzymes [4].

N-acetyltransferase has proven very difficult to purify, partly because of its hydrophobicity, but primarily because its activity appears to be dependent on protein concentration, *i.e.*, activity is eventually lost as the purity of the preparation increases [8]. However, based on [^14^C]acetyl-CoA labeling experiments (which are equivocal, because they assume the formation of E′, see above), the enzyme has been reported to be a dimer of 120 kDa subunits containing Asn-linked oligosaccharides [8]. The 120 kDa subunit has also been postulated to be only the catalytic subunit of a protein complex whose other unidentified members are also needed for functionality [8]. After cloning of the cDNA encoding the transferase, several groups introduced various C-terminal epitope tags as a means of studying the biosynthesis and structure of the enzyme. These tags were shown not to interfere with the intracellular transport or function of the transferase. Multiple bands with varying intensities, corresponding to 120 and 75 kDa [6], or 77, 67 and 48 kDa [16], were detected by Western blot utilizing antibodies directed at the C-terminal epitope tag. The 77 kDa band was first reported as representing the monomer of the active transferase, while the 48 kDa band was thought likely to be a lysosomal degradation product [16]. However, a more recent publication by the same group reported that the protein associated with the 48 kDa band and another protein, associated with a new 29 kDa band (lacking the C-terminal tag), formed the true subunit of the active transferase oligomer, while the protein associated with the 77 kDa band was now to be considered an inactive precursor polypeptide [13]. This group also initially estimated the size of the native oligomer at 240 kDa [8], but more recently places it at 440 kDa [13].

In this report we optimize the detergent extraction and basic biochemical transferase assay procedures so that the specific activity of N-acetyltransferase does not vary with protein concentration and a fully active enzyme can be purified for analyses. We demonstrate that various amounts of several irreversible aggregates (>70 kDa on Western blots) of this hydrophobic protein can form depending on the method of extraction used prior to SDS-PAGE, or even when the purified protein, in the presence of nonionic detergent, is briefly incubated at 37°C. We re-examine the mechanism and effects of co- (endoplasmic reticulum (ER)) and post- (lysosomal) translational processing of N-acetyltransferase on its intracellular transport and expression of enzyme activity, and determine the native molecular weight of the functional enzyme. Finally using our optimized assay with the purified N-acetyltransferase we re-examine the enzyme's kinetic mechanism of action. Our data lead to conclusions that differ substantially from those recently published from a similar study of the enzyme by Durand et al. [13].

## Material and Methods

### Construction of mammalian expression vectors containing either the human full-length or a soluble N-terminal fragment (Met1-Asn163) of the human N-acetyltransferase, both with an epitope tag (His8 & Flag) in C-terminal position

The genomic sequence of *HGSNAT* contains two potential translation initiation sites differing by 84 bp. Although both have a consensus Kozak sequence, ESTs covering the full-length cDNA are only present for the second site (NCBI reference sequence: NM_152419.2, NP_689632.2). Thus, all cDNA constructs expressing human N-acetyltransferase used in this report used the second start site (short form), except when nucleotides encoding a myc-tag were added to the 5′ end (see below).

The full-length human N-acetyltransferase cDNA was previously sub-cloned in pCMVsport6 (Invitrogen) [4]. This construct was used as template for amplification by PCR with the following primers 5′-CACCGCAGCGGGCAGGCAAG-3′ and 5′-GATTTTCCAAAAAATCTTCTTTCTATAGAGGATGTAGGCAATGAG-3′. The product was then inserted into the mammalian expression vector pcDNA3.1D/V5-His-TOPO® (Invitrogen) in frame with the V5-epitope tag. The fidelity of the cloned cDNA was confirmed by sequencing.

The pcDNA3.1D/V5-His-TOPO® construct was next used as template by GenScript (Piscataway, NJ, USA) to generate two of the three constructs used in the present report. First, a full-length human N-acetyltransferase cDNA with a His8Flag tag in C-terminal position (pcDNA - N-acetyltransferase-His8Flag) was constructed by inserting a sequence encoding eight His and the flag peptide (DYKDDDDK) after Ile635 (at nucleotide sequence C1905) followed by a stop codon. Second a shorter construct encoding the short signal peptide, the first luminal loop of N-acetyltransferase, Met1-Asn163 (Fig. 1B) and a His8Flag epitope tag downstream from C489 (Asn163) followed by a stop codon (pcDNA - N-term N-acetyltransferase-His8Flag) was similarly generated. The two constructs were verified by DNA sequencing.

### Addition of a myc epitope tag in N-terminal position of the pcDNA-N-acetyltransferase-His8Flag construct

Generation of the N-terminally tagged myc-N-acetyltransferase-His8Flag construct was accomplished by adding nucleotides encoding the tag to the previous construct (pcDNA-N-acetyltransferase-His8Flag) through a two steps PCR reaction. The following two forward primers were sequentially used

5′-AAACTCATCTCTGAAGAAGATCTGATGAGCGGGGCGGGCA-3′ and 5′-CCAAGCTTGCCGCCATGGAGCAGAAACTCATCTCTGAAGAAGATCTGATGAGC-3′, with the same reverse primer 5′-CCTCGTGGCCGACATATACCAGAAT-3′. The PCR reactions were performed using HotStar HiFidelity DNA Polymerase (Qiagen) and the following conditions: denaturation (94°C, 15 sec), annealing (57°C, 1 min), and extension (72°C, 2 min) for 35 cycles with a final extension step at 72°C for 10 min. Then the PCR fragment encoding the N-terminal myc tag was digested with HindIII and EcoRI, purified and ligated into the pcDNA- N-Acetyltransferase-His8Flag construct (previously digested with the same restriction enzymes). The insert was verified by DNA sequencing.

### Transient and stable expression of the different human N-acetyltransferase constructs

HeLa cells (from American Type Culture Collection) were grown in alpha–MEM medium supplemented with 10% fetal bovine serum (v/v) (both from Wisent Inc.) and 5% antibiotics (penicillin, streptomycin from Gibco BRL) at 37°C in a 5% CO2 humidified incubator. HeLa cells were previously assayed for their endogenous N-acetyltransferase activity, which was found to be very low (∼3 nmol/h*mg).

Transfections using Lipofectamine 2000 (Invitrogen) were performed at ∼90 to 95% confluency following the manufacturer's instructions. These “transiently” transfected cells were used between 20 and 24 h post-transfection or further processed for selection of stable clones (i.e., “permanently” transfected cells). Selection of HeLa cell clones permanently expressing N-acetyltransferase-His8Flag was done after a three weeks culture period in presence of Geneticin (G418, 400 µg/ml, Invitrogen) started at 24 h post-tranfection. The remaining clones were tested for their enzyme activity and the clone expressing the highest N-acetyltransferase activity was maintained in presence of 400 µg/ml of G418 or kept in liquid nitrogen for further use.

### Extraction of N-acetyltransferase in presence of 1% n-dodecyl ß-D maltoside (DDM extract)

HeLa cells (including cells transfected with the different constructs, and non transfected control cells) were harvested and resuspended in phosphate buffer saline (Wisent Inc.) containing 1% of protease inhibitor cocktail (Sigma-Aldrich) and freeze-thawed 6 times. The soluble fraction (lysate) was discarded after centrifugation (100,000 g for 30 min at 4°C) in a benchtop ultracentrifuge (Beckman Coulter). The membrane pellet was resuspended in TBS buffer (Tris-HCl 50 mM, NaCl 150 mM, pH 7.4) containing 1% DDM (w/v) and protease inhibitor cocktail. After homogenization the membrane suspension was rotated overnight at 4°C to extract membrane proteins. The next day the DDM-extract was centrifuged again as above and the resulting supernatant was termed, the DDM or detergent extract.

### Preparation of liposomes and the determination of the optimum lipid composition and concentration to use in the transferase assay

Anionic and neutral liposomes were prepared essentially as previously described [17] with the modifications reported by Tropak et al. [18]. Briefly, liposomes with different lipid composition were obtained by mixing cholesterol from Sigma-Aldrich (CH, 20 mol%), phosphatidyl choline from the same supplier (PC, 80, 60, or 40 mol%), and phosphatidyl inositol from Avanti Polar Lipid (PI, 0, 20 or 40 mol%) in chloroform, and then dried under a stream of nitrogen gas so that upon rehydration a final lipid concentration of 64 mM could be achieved. The lipid mixture thus obtained was rehydrated in CP buffer (pH 5.5) and then freeze-thawed 10 times using a dry-ice/ethanol bath to ensure solute equilibration between bulk and trapped solutions. After rehydration the 64 mM lipids stocks were serially diluted with CP buffer (pH 5.5), such that 2.5 µl of the needed lipid mixture could be added to N-acetyltransferase assay and produce the desired final lipid concentration.

### Optimization of the enzymatic assay for the determination of N-acetyltransferase activity

We optimized the methodology previously reported by Voznyi Ya et al. [11] to measure the N-acetyltransferase activity by using the following protocol. A master mix was prepared such that 27.5 µL (1 reaction volume) of the mix contained 10 µL of Acetyl-CoA 6 mM (Sigma-Aldrich), 10 µL of Mu-GlcNH_2_ 3 mM (Moscerdam Substrates) in CP buffer (pH 5.5) with 0.25% Triton X-100 (v/v), 5 µL Hex (equivalent to 100 nmol/h of total hex activity, from a human placenta ConA fraction [19]) and 2.5 µL of 16 mM liposomes (20% PI lipids, see above). Aliquots (27.5 µL) were distributed in 96 well plates and 2.5 µL of the detergent extract (see above) or purified sample (see below) of N-acetyltransferase was added to each well to start the enzymatic reaction. The reactions were performed in duplicate or triplicate at 37°C for 15 min-3 h. The time of incubation was adjusted to obtain meaningful fluorescence readings relative to the level of enzyme present. The reactions were stopped by addition of 2-amino-2-methyl-1-propanol 0.1 M, pH 10.8 (MAP, 200 µL). Fluorescence of the 4-methylumbelliferone liberated (MU) was measured with a Gemini EM Microplate Spectrofluorimeter (Molecular Devices Inc.) with the excitation and emission wavelengths set at 365 and 450 nm respectively, and analyzed using the SoftMax Pro Software (USA) coupled to the spectrofluorimeter. Protein concentrations were measured using Pierce BCA (bicinchoninic acid) Protein Assays (Thermo Scientific). Quantification of duplicate assays was obtained from bovine serum albumin (BSA) standard curves according to manufacturer's protocol. Specific activities were calculated from the fluorescence of a MU standard solution and expressed as nmol/h*mg of total protein in the sample.

The following variations in the above protocol allowed us to study the effect of, a) the presence or absence of lipid on the linearity of the N-acetyltransferase assay, b) the composition and concentration of the liposomes added in the reaction, and c) either the protein or lipid environment on the stability of the transferase activity. The effect of added lipid or protein (HSA) on the linearity of the transferase activity was tested by measuring the transferase activity of a DDM extract serially diluted and added to 27.5 µL of master mix which contained either CP buffer pH 5.5, buffer plus 0.25% human serum albumin (HSA) or buffer plus liposomes (20% PI). The effect of liposomes composition and/or concentration on the transferase activity was tested by adding liposomes of different lipid composition at different concentration in the assay using equal amounts of purified N-acetyltransferase. Finally to study the stability of the transferase activity as a function of its protein or lipid environment, an identical amount of affinity purified transferase was mixed with equal volume of either CP buffer (pH 5.5) with 0.25% HSA or 16 mM 20% PI liposomes, and incubated at 37°C. Samples were taken at various time points (0–2.5 h) and placed at 4°C. The transferase activity was then measured in triplicate for each time points using the optimized assay conditions (including lipids). An aliquot of each time point was also analyzed by Western blot using the N-terminal antibody.

### Purification of N-acetyltransferase by affinity chromatography

Cell pellets collected from 16 large tissue culture plates of HeLa cells stably expressing N-acetyltransferase, were used to prepare a large amount of DDM extract (see above). Further purification of the DDM extract was performed by affinity chromatography with mouse anti-Flag M2 affinity gel (beads) (Sigma-Aldrich). The concentration of DDM in the extract was reduced from 1% to 0.2% by addition of TBS (plus protease inhibitor cocktail) and then mixed with 80 µL of the anti-Flag M2 affinity gel (Sigma-Aldrich) suspended in TBS plus 0.2% DDM and incubated overnight on a rocking platform at 4°C. The beads with bound N-acetyltransferase protein were collected in a disposable column (Poly-Prep chromatography column 0.8×4 cm, Gibco BRL). The unbound fraction was collected and non-specifically bound proteins were removed by washing the column with 10 mL of washing buffer (0.02% DDM in TBS). Finally the purified protein was eluted with 150 ng/µL FLAG peptide (Sigma-Aldrich) in TBS plus 0.2% DDM, and 250 µL fractions were collected. The fractions were immediately tested for transferase activity and protein concentration. The fractions displaying the highest transferase specific activity were either used immediately (kinetics experiments or Western blots) or stored at −20°C.

### Cell Lines and tissue Culture Conditions

Primary skin fibroblasts derived from patients with I-Cell or MPS IIIC (homozygous for a mutation the prevents proper mRNA splicing [4]) disease, or an unaffected individual (used as a wild-type (WT) control) were provided by the Hospital for Sick Children Tissue Culture Facility and were grown in alpha–MEM medium supplemented with 10% fetal bovine serum (v/v) (both from Wisent Inc.) and 5% antibiotics (penicillin, streptomycin from Gibco BRL) at 37°C in a 5% CO_2_ humidified incubator.

### Western blots

The different subunits corresponding to the N-acetyltransferase protein were detected by Western blot analysis. Samples were diluted in 4× Laemmli sample buffer (Tris-HCl 62.5 mM pH 6.8, 2% SDS (w/v), 5% ß-mercaptoethanol (v/v), 10% glycerol (v/v) and 0.002% bromophenol blue (w/v)), incubated at room temperature (RT) overnight and resolved by SDS-PAGE using Nu-PAGE 4–12% Bis-Tris gel (Invitrogen). Then proteins were electrophoretically transferred to polyvinylidene fluoride (PVDF) membranes (BioRad), which were then blocked with 5% (w/v) non-fat dry milk in TBST (Tris-HCl 25 mM, pH 7.4, NaCl 137 mM, KCl 2.7 mM and 0.1% Tween-20 (v/v)) overnight at 4°C. After incubation with primary (90 min at RT) and secondary antibodies (1 h at RT), signals were generated using enhanced chemiluminescence (ECL and ECL Advanced western blotting detection, Amersham GE Healthcare). The following primary antibodies were used: rabbit polyclonal IgG anti-DYKDDDDK (*i.e.*, Flag) Tag and anti-EEA1 (an early endosomal marker) from Cell Signaling Technology at 1/1000 dilution; goat (sc-6465) anti Calnexin (an ER marker) from Santa Cruz Biotechnology at 1/200; mouse monoclonal Flag M2 IgG from Sigma-Aldrich at the same dilution; rabbit polyclonal HGSNAT IgG (raised against peptide corresponding to Q52 to N156 of N-acetyltransferase protein) from Sigma-Aldrich at 1/1500; mouse monoclonal myc IgG (clone 4A6) from Millipore at 1/1000; rabbit polyclonal Hex A IgG at 1/400 and rabbit polyclonal cation-independent mannose phosphate receptor IgG at 1/1000 dilution, the last 2 antibodies were produced in our laboratory. The secondary antibody used were either HRP-conjugated donkey anti-rabbit, donkey anti-goat (Jackson Immunology) or goat anti-mouse at 1∶10,000 dilution (Santa Cruz). Samples were analyzed by Western blot were processed as indicated above, unless otherwise noted in the figure legend.

### SYPRO Ruby staining

Protein concentration of affinity purified N-acetyltransferase was estimated using SYPRO Ruby methodology from Molecular Probes (Invitrogen) to eliminate the interference of the Flag peptides used to elute the enzyme. In short, a sample of purified N-acetyltransferase was separated by SDS-PAGE on a Nu-PAGE 4–12% Bis-Tris gel (Invitrogen) in parallel with increasing amounts of BSA (0–800 ng). After fixation in a methanol (50%) - acetic acid solution (7%) for 15 min at RT, the gel was stained using the SYPRO Ruby protein gel stain solution for 30 min (including 3×30 sec microwave period) and washed with a methanol (10%) - acetic acid (7%) solution for 30 min at RT. The luminescence of SYPRO ruby was recorded using a phosphorimager (Molecular Dynamics, Storm 840 with ImageQuant software) and the pixel intensities corresponding to the sum of the 2 luminescent bands observed for BSA was plotted against their concentrations to generate a standard curve. The protein concentration in the N-acetyltransferase sample was calculated from the sum of the pixel intensities measured from the 3 major luminescent bands observed on the gel migrating at 62, 44 and 27 kDa respectively.

### Edman-Amino acid sequencing

Amino acid sequencing was performed by the Advanced Protein Technology Center at the Hospital for Sick Children (http://www.sickkids.ca/Research/APTC/Edman-Sequencing/index.html). In short, purified N-acetyltransferase blotted onto PVDF membrane was excised and treated with phenylisothiocyanate. The produced cleavable phenylthiohydantoin amino acid derivatives were subjected to N-terminal sequencing by Edman degradation using Procise 490 Protein Sequencer, the Model 140 C Microgradient Delivery System. Data quantitation and protein sequencing reporting was generated using Procise control software and the Model 610A Data Analysis software.

### Purification of iron-dextran-filled lysosomes by magnetic chromatography (FeDex)

N-acetyltransferase transfected HeLa cells were grown in presence of paramagnetic iron colloidal particles for 9 hours [20]. After several washes cells were gently homogenized and post-nuclear supernatant (PNS) collected at 2,000 rpm at 4°C. An enriched lysosomal fraction was obtained by magnetic separation of iron-dextran-loaded lysosomes from the PNS by using MACS separation columns (Miltenyi Biotec) as previously described [21].

### Endo H and PNGase treatment

The glycosylation state of N-acetyltransferase-His8Flag from stably transfected Hela cells was analyzed by using purified lysosomal fractions (above). The glycosylation state of myc-N-acetyltransferase-His8Flag from transiently transfected Hela cells was analyzed using a DDM extract (as described above). And finally the glycosylation state of N-term N-acetyltransferase-His8Flag was analyzed using the soluble fraction obtain after freezing-thawing cell pellet (lysate) from HeLa cells transiently transfected with the N-terminal construct. The different samples were incubated in the presence or absence of Endo H endoglycosidase and PNGase glycopeptidase (New England BioLabs) according to the manufacturer's protocol. Denaturation of the full-length glycoproteins was performed at 37°C for 2 hours or for the soluble N-terminal fragment, by boiling. EndoH or PNGase was then added and the deglycosylation reaction carried out at 37°C for either 4 hours, for the N-acetyltransferase protein because of its TMDs, or only 2.5 hours for the N-terminal fragment. Treated and control samples were subjected to Western blot analysis as described above using antibodies as indicated in figure legend.

### Immunoprecipitation

DDM extracts from HeLa cells transiently transfected with the myc-N-acetyltransferase-His8Flag construct or permanently transfected with the control N-acetyltransferase-His8Flag construct were diluted to 0.2% DDM, and myc mouse monoclonal IgG added. The reactions were gently mixed for 1 h at 4°C followed by the addition of 50 µL of 50% slurry of washed GammaBind Plus Sepharose (GE Healthcare) (protein G beads). The mixtures were rotated overnight at 4°C. The N-acetyltransferase activities immobilized on the Sepharose beads and that remained in unbound fractions were measured separately after a centrifugation and washing steps (as above).

### Indirect immunofluorescence staining and confocal microscopy imaging

Indirect immunolabeling was performed using a previously described protocol [22] using transiently transfected HeLa cells at 20 h post transfection with either pcDNA-N-acetyltransferase-His8Flag, pcDNA-N Term N-acetyltransferase-His8Flag or pcDNA-myc-N-acetyltransferase-His8Flag construct (untransfected cells represent “in situ” controls).

Primary antibodies were as follows: Mouse Flag (M2) IgG and human glucocerebrosidase rabbit polyclonal IgG [21], or a N-terminal N-acetyltransferase rabbit polyclonal and a protein disulfide isomerase (PDI) mouse monoclonal IgG (Stressgen). Secondary antibodies were chicken anti-mouse Alexa fluor 488 (green) for flag and goat anti-rabbit Alexa fluor 594 (red) for glucocerebrosidase or chicken anti-rabbit Alexa 488 (green) for the N-terminal of N-acetyltransferase and goat anti-mouse Alexa 594 (red) for PDI (Molecular Probes) at a 1∶200 dilution in blocking solution. Samples were analyzed using a Zeiss Axiovert confocal laser microscope equipped with a 63×1.4 numerical aperture Apochromat objective (Zeiss) and LSM 510 software. DAPI-stained nuclei were detected on the same system with a Chameleon two-photon laser. Confocal images were imported and contrast/brightness adjusted using Volocity 5 program (Improvision Inc.).

### Native size exclusion chromatography column

DDM extracts (∼600 µL) from 2 large tissue culture plates of N-acetyltransferase transfected HeLa cells were prepared as above. The resulting membrane extract (∼500 µL) was then loaded onto a Sephacryl S-400 HR column (total column volume 153 mL, Pharmacia Biotech) with TBS plus DDM 0.02% as mobile phase. The column calibration was performed using the following proteins as molecular weight standard thyroglobulin (669 kDa), alcohol dehydrogenase (150 kDa), bovine serum albumin (66 kDa), ovalbumin (45 kDa) and carbonic anhydrase (29 kDa), and blue dextran and sodium azide were used as markers for void volume and total volume respectively (all from Sigma-Aldrich). Elution was performed under gravity at 4°C with 1 mL fractions collection. Each fraction was assayed for N-acetyltransferase activity (aliquots of 2.5 µL as described above). Protein concentration for each fraction was also determined and every other fraction between elution the volume of 70 mL to 160 mL were analyzed by dot blot (only every 5 fraction is shown under the graph). An aliquot (6 µL, 3×2 µL) was applied onto a nitrocellulose membrane (Protran BA 85, Whatman) and processed similarly to a western blot (see above) using polyclonal rabbit N-terminal N-acetyltransferase IgG as the primary antibody.

### Kinetic analyses

Kinetic analyses were performed using the optimized N-acetyltransferase assay with freshly affinity purified transferase. Because the concentrations of the 2 substrates were varied between the different kinetics experiments, a slightly different protocol was used for the transferase assay. Instead of preparing a master mix with all of the reagents then adding the transferase as indicated earlier, we prepared a mix containing multiples of fixed amount of purified transferase (2.5 µL), liposomes (2.5 µL) and Hex (5 µL), corresponding to 10 µL per reaction, and then added the 2 substrates separately allowing us a great flexibility in their respective concentration.

To generate the Michaelis-Menten and Lineweaver-Burk plots either the final concentration of the substrate Mu-GlcNH2 ranged between 0.01 to 1 mM while the Acetyl-CoA concentration was kept fixed at 0.17, 0.33 or 1.0 mM, or the final concentration of Acetyl-CoA ranged from 0.0833 to 2 mM while the Mu-GlcNH2 concentration was kept fixed at 0.05, 0.10 or 0.30 mM.

To determine the kinetic parameters, the concentration of MU-GlcNH2 ranged between 0.01 and 0.5 mM while the Acetyl-CoA concentration was constant at 2 mM (saturating). Or the concentration of Acetyl-CoA ranged between 0.0833 and 2 mM while MU-GlcNH2 was constant at 1 mM (saturating). The values of the kinetic parameters (Km and Vmax) were extracted via nonlinear regression analysis using Prism 5.0 (Graph Pad Software. Inc., USA). Kcat were then separately calculated from the respective Vmax. The standard errors were calculated from the best-fit curves generated by the computer program.

### Determining if a acetylated protein intermediate (E′) is formed when purified N-acetyltransferase is incubated with [^3^H]acetyl-CoA

DDM extracts were prepared from 2 large tissue culture plates of N-acetyltransferase permanently transfected and untransfected (control) HeLa cells. The same amount of protein from both samples was diluted with TBS to achieve a final concentration of 0.2% for DDM. After addition of 40 µL of pre-equilibrated (in TBS plus DDM 0.2%) anti-flag M2 affinity gel suspension, the samples were rotated overnight at 4°C. The following day, the beads were washed 3 times with TBS plus DDM 0.02%, and incubated with 0.6 µCi of [^3^H] acetyl-CoA (2.5 Ci/mmol, Perkin Elmer) in CP buffer either at pH 6.5 or pH 6.8 containing 0.1% DDM, in presence or in absence of liposomes (20% PI, final concentration 1.33 mM) at RT for 1 or 0.5 h, respectively. After centrifugation and three washes with TBS plus 0.02% DDM, the beads and unbound fraction (including the washes) were collected separately and their radioactivity measured by liquid scintillation. Three independent experiments for stably N-acetyltransferase transfected and HeLa control were performed. Using the same experimental conditions another set of experiments was performed in which [^3^H] acetyl-CoA was omitted to allow for the determination of the amount of N-acetyltransferase activity that was immobilized.

## Results

Classic extraction with Triton X-100 [6], [11], [23], [24] of N-acetyltransferase from a HeLa cell line, established from a clonal population of cells permanently transfected with the enzyme containing a 2.1 kDa C-terminal-His8Flag tag (Fig. 1A), was compared to extractions with other detergents. It was found that the transferase specific activity in 0.1% or 1% Triton, or in 0.1% NP-40 extracts was 130–170 nmol/h*mg. On the other hand extraction with 1% n-dodecyl ß-D maltoside (DDM) resulted in a specific activity of 530 nmol/h*mg. The transferase activity in the DDM-extract also remained stable for at least 6 months at -20°C.

**Figure 2:**
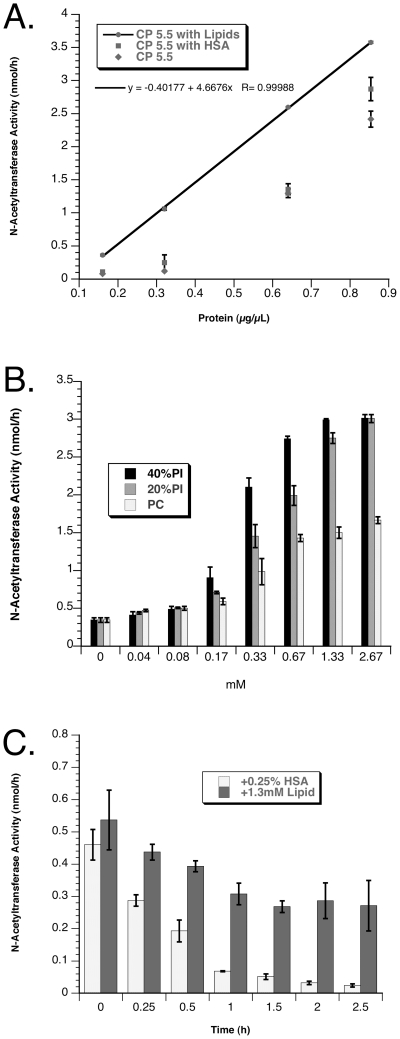
Effects of lipids on N-acetyltransferase activity are illustrated. Each set of data points represents the average of triplicate determinations of N-acetyltransferase activity with their standard deviations (SD) shown as error bars (A) DDM extracts from permanently transfected HeLa cells were serially diluted in CP buffer, pH 5.5 (filled diamonds), CP buffer containing 0.25% HSA (filled squares) or CP buffer containing 1.3 mM 20% PI (filled circles with the error bars representing SD too small to see, connected by the best fit line, R = 1) (X-axis, total extracted protein; Y-axis, transferase activity in nmol/h). (B) Equal amounts of anti-Flag-column purified N-acetyltransferase were assayed in the presence of increasing concentrations of lipids (X-axis, mM) with decreasing mole% of negatively charged PI (40-0% PI+40–80% PC+20% CH); 40% PI, darkly shaded bars; 20% PI, grey hatched bars; or PC (0% PI), white bars. (C) Stability of identical amounts of purified N-acetyltransferase in CP buffer pH 5.5, containing either 0.25% (w/v) HSA (white bars) or 1.3 mM, 20% PI, lipids (shaded bars) (X-axis, hours at 37°C; Y-axis transferase activity in nmol/h).

We investigated the linearity of the classic fluorescent-based, coupled transferase assay [11], [24] using N-acetyltransferase transfected HeLa cells extracted with DDM. The activity dropped precipitously with little activity remaining after the total extracted protein concentration was reduced from 0.65 to 0.33 µg/µL, even when 0.25% BSA (w/v) was included in the dilution buffer (Fig. 2A). Since *in vivo* the N-acetyltransferase resides in the highly negatively charged environment of the lysosomal membrane, we investigated the possibility that its transferase activity was dependent on the presence of negatively charged lipids, rather than other unidentified proteins, as previously suggested [8]. N-acetyltransferase, purified on anti-Flag beads, was assayed in the presence of neutral or anionic liposomes, prepared and used essentially as was done for GM2 ganglioside assays with the GM2 Activator protein and ß-hexosaminidase (Hex) A [17]. An increasing negative charge was obtained by increasing the content of PI with a commensurate decrease in the level of neutral PC. The addition of negatively charged lipids to the assay resulted in a dramatic increase in N-acetyltransferase activity with the optimal activity observed with either 1.3 mM of lipid containing 20 or 40% PI, or 0.67 mM containing 40% PI (Fig. 2B). A comparison of the stability of the purified enzyme at 37°C in CP pH 5.5, containing 0.1% DDM and either HSA (0.25%, w/v) or lipid (1.3 mM, 20% PI) demonstrated that the presence of lipids also greatly enhanced the enzymes resistance to heat inactivation (Fig. 2C). These data demonstrate that in the absence of lipids the purified protein loses ∼50% of its transferase activity after only 0.5 h at 37°C. The addition of the lipid mixture to the buffer used to dilute the original DDM extract from transfected HeLa cells also preserved the linearity of the transferase reaction below total protein levels of 0.6 µg/µL (Fig. 2A). The reaction remained linear for at least 3 h (data not shown). Levels of Hex needed in the coupled reaction were also evaluated. It was determined that 100 units (nmol MUG/h) of Hex per assay were sufficient to ensure linearity even at very high levels of transferase activity (data not shown).

**Figure 3:**
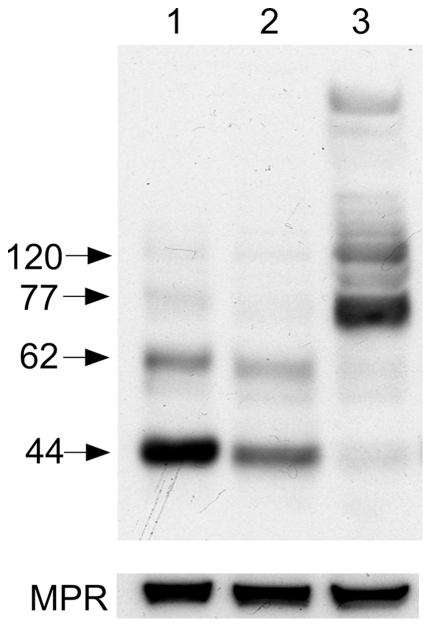
The effects of detergent extraction procedures on the intensity of bands associated with monomeric N-acetyltransferase (i.e., 62 and 44 kDa), detected by Western blotting with the C-terminal rabbit Flag antibody. HeLa cells, permanently transfected with N-acetyltransferase-His8Flag, were; lane 1- first extracted with 1% DDM, centrifuged and the extract denatured in SDS-PAGE sample buffer (containing a reducing agent) at room temperature; lane 2- homogenized (in water), sonicated and boiled in lithium dodecyl sulfate reducing sample buffer [13]; or lane 3- extracted with 2% SDS, diluted with SDS-PAGE reducing sample buffer and heated at 65°C [6]. Bottom panel shows the loading control, the cation-independent mannose-6-phosphate receptor, which contains a single TMD.

Many proteins with multiple TMDs denature during and after extraction by forming irreversible aggregates. Extracting and heating samples in SDS prior to PAGE can also enhance the formation of these aggregates [25]. Thus we examined the effects on the banding pattern produced from reduced samples on Western blots (visualized with a Flag antibody) using N-acetyltransferase extracted from transfected HeLa cells. We compared our method of first extracting with 1% DDM then denaturing with sample buffer (2% SDS plus reducing agent) at RT (Fig. 3, lane 1), with other previously published methods. These included homogenized, sonicated, and then boiled in lithium dodecyl sulfate reducing sample buffer (Invitrogen) [13] (Fig. 3, lane 2) or extracted with 2% SDS followed by dilution with SDS-PAGE reducing sample buffer and heating at 65°C [6] (Fig. 3, lane 3). Whereas the method used in this report, initial DDM extraction, produced the highest yield of unaggregated N-acetyltransferase, direct extraction with 2% SDS primarily produced irreversible aggregates. The major aggregate had an apparent Mr of 77 kDa followed by a secondary aggregate band at 120 kDa (Fig. 3, lane 3). Thus we concluded that after SDS-PAGE any bands observed for our N-acetyltransferase construct, with its small 2.1 kDa epitope tag (Fig. 1A) >70 kDa represent aggregates.

**Figure 4:**
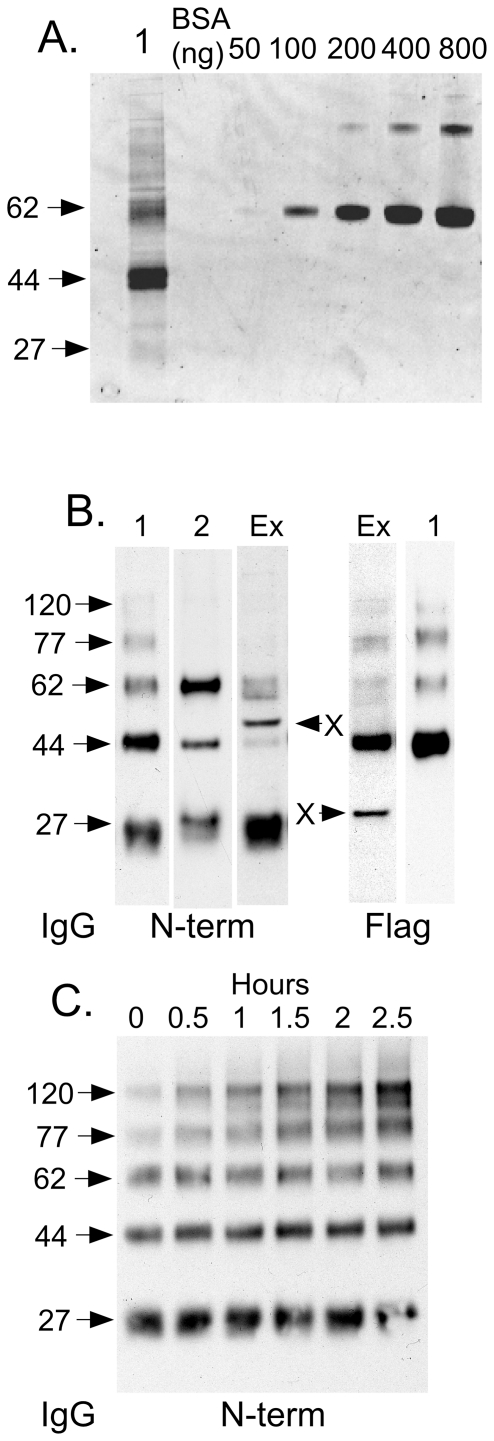
Purification of N-acetyltransferase by anti-Flag affinity chromatography. (A) Lane 1- SDS-PAGE separation of the purified enzyme under reducing conditions detected with fluorescent SYPRO Ruby protein stain. Also shown is a dilution series of BSA used to calculate the protein levels in the transferase sample and the specific activity of the purified enzyme; and (B) Western blotting using either an antibody against the Gln53-Asn156 epitope (N-term), or against the C-terminal Flag tag (Flag). Lane-1 contains the transferase that was bound, eluted with Flag peptides and then denatured, while lane 2 contains enzyme that was directly released and denatured from the anti-Flag column with SDS sample buffer (containing a reducing agent). Aliquots of the initial 1% DDM extract (Ex) were also examined by Western blotting. Bands marked “X” in the Ex lanes are non-specific proteins detected by the antibodies (the ∼30 kDa X band is only seen with the rabbit Flag antibody). (C) The effects of incubating the purified protein at 37°C for 0–2.5 h without added lipids were examined by Western blotting using the N-terminal antibody.

N-acetyltransferase was purified from 16×15 cm plates of transfected HeLa cells using an anti-Flag affinity column, eluted with Flag peptides. The components of the purified protein preparation were separated under reducing conditions by SDS-PAGE and visualized using SYPRO Ruby protein gel stain. Two dominant bands at 62 kDa and 44 kDa, also seen on Western blots of the DDM extract (Fig. 3), plus a light band at 27 kDa (Fig. 4A) were detected. Western blots of the purified protein, using anti-Flag IgG (C-terminal antibody) or a commercially available antibody made against residues 53–156 (Fig. 1) of N-acetyltransferase (N-terminal antibody), were compared to the protein stain (Fig. 4B). Both antibodies detected the 62 kDa band. The 44 kDa band was strongly recognized by the C-terminal and weakly by the N-terminal antibody. The 27 kDa band was strongly recognized only by the N-terminal antibody. These data suggest that the larger band corresponds to the full length polypeptide and that the 27 and 44 kDa bands correspond to the N-terminal and C-terminal portions of the 62 kDa precursor polypeptide, respectively, generated by a posttranslational proteolytic event(s). Interestingly the ratio of intensities of these bands varied not only as a function of the antibody used, but also with the sample preparation method. For example t`he 62 kDa band was more intense when the N-terminal antibody was used and the protein was directly eluted from the Flag column using SDS sample buffer (Fig. 4B lane 2), rather than first being eluted with Flag peptides and then denatured in SDS sample buffer (Fig. 4B lane 1). While in the former case aggregate bands were not detectable, in the latter case two aggregate bands at 77 kDa (major) and 120 kDa were clearly visible. On the other hand with the N-terminal antibody, the crude cell extract produced the most intense 27 kDa band and a very weak 44 kDa band (Fig 4B, left panel, lane “Ex”). These variations may result from differences in the antibody accessibility to the associated protein-epitope. It also follows that the small 27 kDa protein is poorly stained by the SYPRO Ruby protein stain as compared to the proteins associated with the 62 or 44 kDa bands (Fig. 4A). The effects on the banding patterns produced by Western blots utilizing the N-terminal antibody were investigated after incubation of the purified enzyme in the absence of added lipids at 37°C over time (0–2.5 h). The resulting Western blot demonstrated that along with a rapid loss of activity (Fig. 2C) there is also a time dependent increase in the levels of irreversible aggregate bands, particularly the 77 and 120 kDa species (Fig. 4C).

The final specific activity of the affinity purified N-acetyltransferase in the presence of lipid was estimated at 260 µmol MU/h*mg with a yield of ∼50% and an enrichment of ∼500-fold over the DDM-extract. Because of the presence of the Flag peptides used to elute N-acetyltransferase-His8Flag, protein concentration was estimated from the relative fluorescence units of the Ruby stained gel, using a dilution series of bovine albumin as a protein standard (Fig. 4A, BSA). Importantly, these data indicate that the protein encoded by the *HGSNAT* gene alone is sufficient to produce N-acetyltransferase activity, *i.e.*, N-acetyltransferase is not a multimer of two or more subunits encoded by different genes as has been previously suggested [8].

The mature amino-termini of the N-acetyltransferase alpha- and ß-polypeptides were determined by Edman degradation of the proteins contained within the excised bands from the above gel (Fig. 4A). The amino terminal sequence obtained from the alpha-chain was KKRHAE48 and from the ß-chain, GVSEIA150 (Fig. 1A). These data are consistent with the specificity of the two antibodies used in the Western blots (12 N-terminal residues of the ß-chain were part of the peptide epitope used to generate the N-terminal antibody). Edman degradation analysis of the 62 kDa band failed to produce any sequence data, suggesting the presence of a blocked amino terminus. Mass spectrometry analysis of the tryptic peptides generated from the 62 kDa band demonstrated the presence of peptides predicted to be part of both the alpha- and ß-chains, consistent with its recognition by both antibodies and indicative of it being the N-acetyltransferase precursor polypeptide. However no peptides were identified that corresponded to the predicted signal peptide (data not shown).

**Figure 5:**
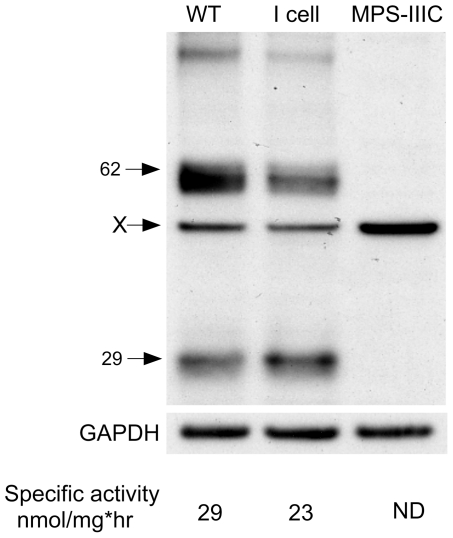
Western blot analysis of the endogenous N-acetyltransferase protein in three human fibroblast cell lines. Fibroblast from an unaffected individual (WT), a patient with I-Cell disease, and a MPS IIIC patient were extracted with 1% DDM, 20 µg each of extracted protein were separated by SDS-PAGE, and the N-acetyltransferase proteins visualized using the N-terminal antibody. The blot was reprobed with anti GAPDH as a loading control. The specific activity of each extract is shown at the bottom, ND is not detected.

Since the new commercially available N-terminal antibody offered us the opportunity to probe the structure of endogenous N-acetyltransferase in human fibroblast, we confirmed the presence of the 62 kDa precursor and 27 kDa processed alpha-chain (the alpha-chain from human fibroblast migrates at an apparent Mr of 29 kDa) in normal and I-cell patient fibroblasts, and confirmed the lack of these bands in an extract from a MPS IIIC patient's cells (Fig. 5) homozygous for a mutation that prevents normal mRNA splicing [4]. I-cell disease fibroblasts lack GlcNAc-1-phosphotransferase and are unable to tag soluble lysosomal enzymes and proteases with mannose-6-phasphate residues [26], [27], which are required for targeting them to the lysosome via the mannose-6-phosphate receptor [28]. Despite being deficient in many soluble proteases no increase in the levels of the 62 kDa band versus the processed 27 (29) kDa band was seen, nor was the specific activity of N-acetyltransferase significantly decreased (Fig. 5). The 44 kDa band was also detected, but required much longer exposure times, causing the 62 and 27 (29) kDa bands to be severely over-exposed (data not shown).

**Figure 6:**
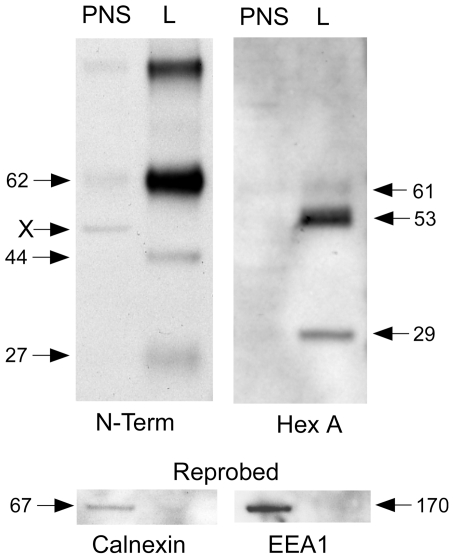
Western blot analysis of the post nuclear supernatant (PNS) and enriched lysosomal (Lys) fractions separated magnetically from extracts of N-acetyltransferase-His8Flag permanently transfected HeLa cells loaded with iron-dextran (FeDex). After SDS-PAGE the proteins in the PNS and Lys fractions (1 µg total protein from each fraction was loaded) were visualized with the N-terminal N-acetyltransferase antibody (N-term). The nonspecific ∼50 kDa band visualized with the N-terminal antibody is marked as “X”. As control for the enrichment of lysosomes in the “Lys” fraction, the blot was stripped and re-probed with an antibody against human lysosomal ß-hexosaminidase A (Hex A). Furthermore, markers for the ER (Calnexin) and early endosome (EEA1) were visualized after stripping and re-probing the blot with the appropriate antibody (bottom).

The suspected lysosomal cellular location for processing of the precursor chain was confirmed by magnetic fractionation of N-acetyltransferase from transfected cells loaded with iron dextran particles (FeDex method) [29]. Cell homogenates were separated into a post-nuclear-supernatant (PNS) and an enriched lysosomal (Lys) fraction, and equal amounts of total protein (1 µg) were analyzed by Western blotting (Fig. 6). A Western blot for N-acetyltransferase-His8Flag protein was produced using the N-terminal N-acetyltransferase antibody (Fig. 6, N-term). Much larger amounts of the precursor form of N-acetyltransferase was detected in the lysosomal (Lys), as compared to the PNS fraction of cells. However the processed 44 kDa ß-and 27 kDa alpha- chains were only detected in the Lys fraction. An unidentified contaminant band at ∼50 kDa (also seen in Fig. 4B) was also detected in the PNS fraction (Fig. 6, X). As a positive control for the enrichment of lysosomes in the Lys-fraction, the blot was stripped and re-probed with an IgG against the lysosomal marker Hex A (Fig. 6, Hex A). The expected 53 (alpha-subunit) and 29 kDa (ß-subunit) bands corresponding to the processed mature forms of the Hex A subunits were only detected in the Lys fraction [30]. The two ∼61 kDa alpha- and ß- precursor chains were weakly detected in both fractions [30]. Reprobing of the blot with IgG against the ER marker calnexin and the early endosomal marker EEA1 demonstrated that these proteins were concentrated in the PNS fraction of cells (Fig. 6, bottom). These results indicate that the single 62 kDa N-acetyltransferase precursor chain is efficiently transported to the late endosome/lysosome after synthesis where it is processed into its mature alpha- and ß-chains.

**Figure 7:**
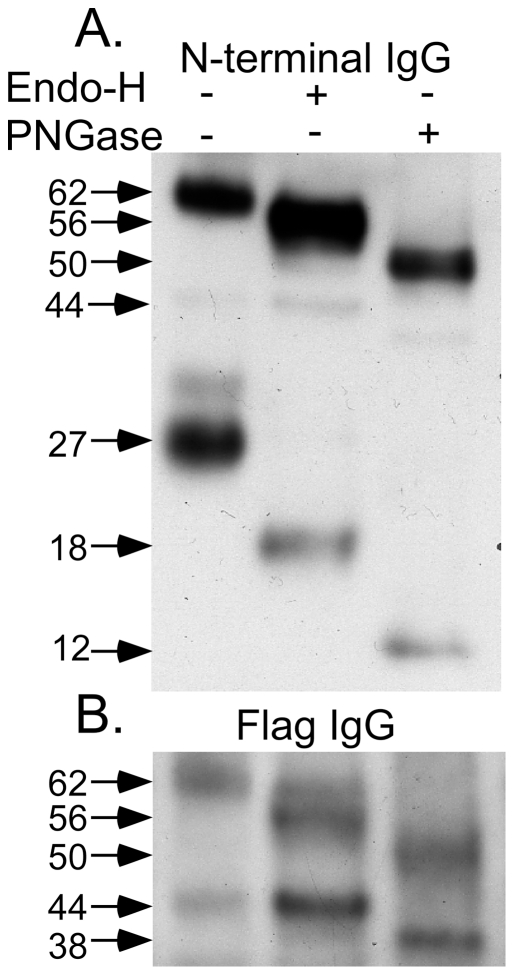
Western blot analyses of the N-acetyltransferase-His8Flag protein from the “Lys” fraction of **Figure 6** (lane 1) treated with either endo-H (lane 2) or PNGase (lane 3). Proteins were detected with either, (A) the N-terminal N-acetyltransferase antibody or (B) the Flag M2 antibody.

The oligosaccharide structures of the proteins contained in the three major bands associated with N-acetyltransferase in the above lysosomal enriched fraction (free of the confounding ∼50 kDa nonspecific band, Fig. 6 “X”) were examined as substrates for endoglycosidase H (endo-H) and/or glycopeptidase-F (PNGase) digestions (Fig. 7A & B). The 62 kDa precursor band contained both endo-H sensitive (high mannose type) and insensitive (complex type) oligosaccharides. The N-terminal alpha-chain, predicted to have 3 potential glycosylation sites (Fig. 1), also contained both types of oligosaccharides (Fig. 7A), while the ß-chain, predicted to have 2 potential glycosylation sites (Fig. 1) contained only complex types (Fig. 7B). Furthermore, the Mr calculated from the gel of the mature, deglycosylated alpha-chain was 12 kDa (Fig. 7A), which is nearly identical to the 11.7 kDa predicted for residues Lys43-Asn144 from N-acetyltransferase, and significantly different from the 16 kDa predicted for the alpha-chain retaining the signal peptide (Fig. 1A).

The N-acetyltransferase precursor protein has previously been reported to be inactive and to have retained its signal peptide [13]. This would result in the signal peptide becoming the first of 12 permanent TMDs. However, it is clear from the N-terminal sequence (13 residues C-terminal to the predicted signal peptide cleavage site (Fig. 1A)) and the observed Mr of the deglycosylated alpha-chain (Fig. 7A) that if the signal peptide was not cleaved co-translationally by the signal peptidase in the ER, then it would be removed postranslationally during the lysosomal maturation of N-acetyltransferase, resulting in a protein with 11 TMDs.

**Figure 8:**
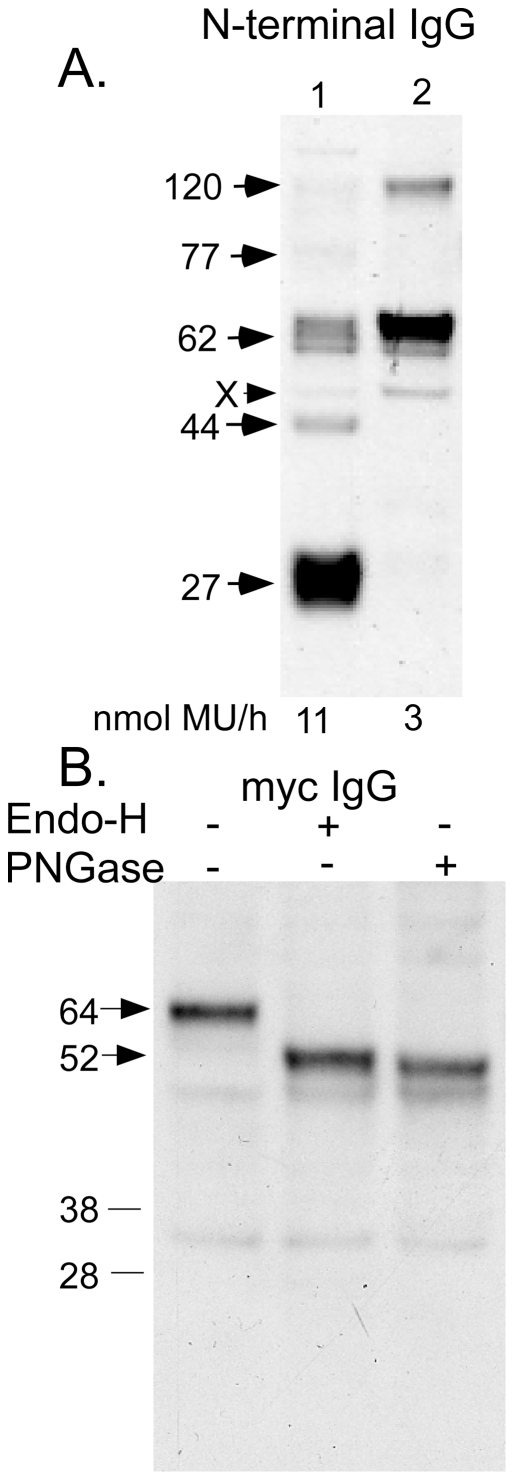
The protein expressed by a construct encoding myc-N-acetyltransferase-His8Flag was analyzed by Western blotting. (A) Extracts from HeLa cells permanently expressing the singly-tagged N-acetyltransferase-His8Flag (lane 1) were compared with HeLa cell transiently expressing myc-N-acetyltransferase-His8Flag (lane 2) using the N-terminal N-acetyltransferase antibody. The levels of transferase activity in the extracts are given below each lane. (B) The oligosaccharides present on the doubly-tagged myc-N-acetyltransferase-His8Flag protein (lane 1) were analyzed based on endo-H (lane 2) and/or PNGase (lane 3) sensitivities by Western blotting using a myc antibody.

**Figure 9:**
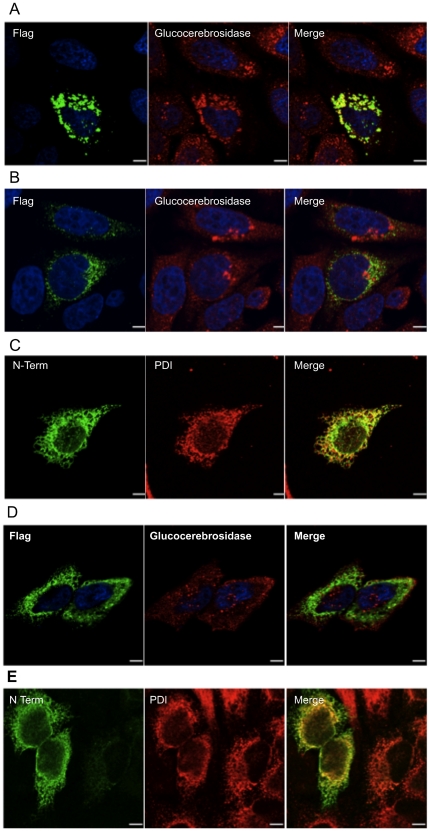
Indirect immunofluorescence staining and confocal microscopy imaging of HeLa cells transiently expressing various forms of N-acetyltransferase. (A) N-acetyltransferase-His8Flag, (B & C) myc-N-acetyltransferase-His8Flag, or (D & E) the N-terminal segment (Met1-Asn163-His8Flag) of N-acetyltransferase (Fig. 1). Cells were stained with the mouse M2 Flag antibody (green A, B & D) and a rabbit antibody against either a lysosomal marker, glucocerebrosidase (red, A, B & D), or against the N-terminus of N-acetyltransferase (green) and a mouse antibody against a ER marker, PDI (red, C & E), and the images merged. Scale bars represent 10 µm. Images shown are representative of 10 recorded.

In order to clarify the N-terminal structure of the N-acetyltransferase precursor, a construct encoding a myc-tagged extension (10 residues) to the N-terminus of the signal peptide, was transiently expressed in HeLa cells. Western blot analysis comparing this doubly tagged protein with singly-tagged N-acetyltransferase-His8Flag (using the N-terminal antibody) revealed the presence of a single major band (Fig. 8A lane 2) corresponding to a slightly higher Mr than the singly-tagged 62 kDa precursor (Fig. 8A, lane 1). Significantly, no band corresponding to the processed alpha-chain was detected in the myc-N-acetyltransferase-His8Flag sample (lane 2). The specific transferase activities of the cell extracts were also determined, with untransfected HeLa cell extract containing 3 nmol/h*mg of endogenous activity. The extract from the permanently transfected cells containing the singly-tagged protein was ∼4-fold greater (540 nmol/h*mg) than the cell extract containing the transiently expressed doubly tagged N-acetyltransferase (150 nmol/h*mg). This difference is consistent with the levels of N-acetyltransferase cross-reacting material detected on the Western blot (Fig. 8A). These data demonstrate that despite a lack of processing, the doubly-tagged protein is fully active. This conclusion was further confirmed after immuno-precipitation of extracts from the two cell lines with myc IgG immobilized on Protein-G beads. The immobilized transferase activity from the doubly-tagged (+myc) protein extract was 12 nmol/h, as compared to 0.7 nmol/h for the singly-tagged (-myc) protein extract. These data also demonstrate that the extended myc-tagged signal peptide is retained in the active doubly-tagged precursor protein. Next the oligosaccharide structures on the myc-N-acetyltransferase-His8Flag were examined and found to be fully endo-H sensitive, i.e., it contained only high mannose type oligosaccharides, indicating an ER localization (Fig. 8B). Indirect immunofluorescence and confocal microscopy imaging were used to compare the cellular localization of the singly- (Fig. 9A) and doubly- (Fig. 9B & C) tagged N-acetyltransferase proteins using the Flag or N-terminal antibody. The protein with only the C-terminal tag co-localized with the lysosomal marker ß-glucocerebrosidase (Fig. 9A), while the doubly tagged protein did not (Fig. 9B), again consistent with an ER-localization. This was confirmed by using an IgG against the ER marker PDI, which co-localized with the doubly- tagged enzyme (Fig. 9C). These data suggest that the retention of the signal peptide prevents intracellular transport of the precursor to the lysosome and proteolytic processing into the mature alpha- and ß- chains, but does not prevent it from expressing transferase activity.

**Figure 10:**
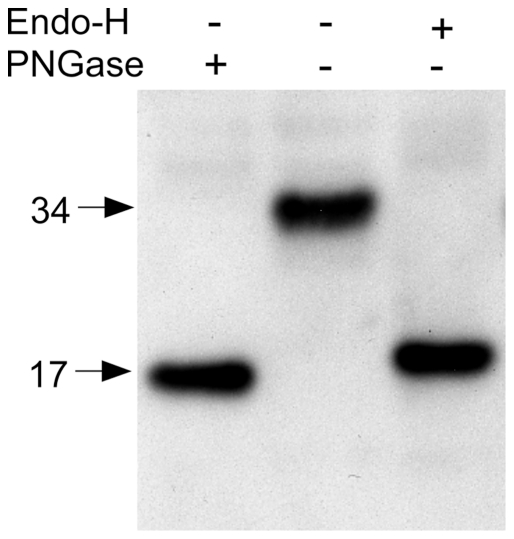
The degree of endo-H versus PNGase sensitivities was used to probe the level of Asn-linked oligosaccharide processing that occurs on the N-terminal fragment (Met1- Asn163-His8Flag) of N-acetyltransferase (**Fig. 1**) from transiently expressing HeLa cells. Proteins from soluble cell lysates (i.e., not detergent extracts) were analyzed by Western blotting using the N-terminal antibody.

In order to determine if the wild-type signal peptide was co-translationally cleaved, an N-acetyltransferase N-terminal construct, encoding residues 1–163-His8-Flag (stopping 2 residues short of the first TMD, Fig. 1B), was made and transiently expressed in HeLa cells. This protein should lack all signals for lysosomal incorporation. The resulting protein (without oligosaccharides) would have a predicted Mr of 19.6 kDa with and 17 kDa without the signal peptide (Fig. 1B). The protein was not secreted, but it was detected, along with other soluble enzymes, in the detergent-free lysate of the transfected cells. This observation suggests that the protein is retained in the ER, but without its signal peptide, which would have acted as a TMD. Immunofluorescence images confirmed that the N-terminal fragment was not located in the lysosome (Fig. 9D), but in the ER (Fig. 9E). The protein was analyzed by Western blotting with the N-terminal antibody. A single band was detected with a Mr of 34 kDa (Fig. 10, lane 2). Significantly, the fragment was also found to contain only endo-H sensitive oligosaccharides, consistent with an ER localization, and after deglycosylation it was associated with a 17 kDa band (Fig. 10. lanes 1 & 3). From these data we conclude that the signal peptide is co-translationally cleaved in both the N-terminal fragment and in full length N-acetyltransferase.

**Figure 11:**
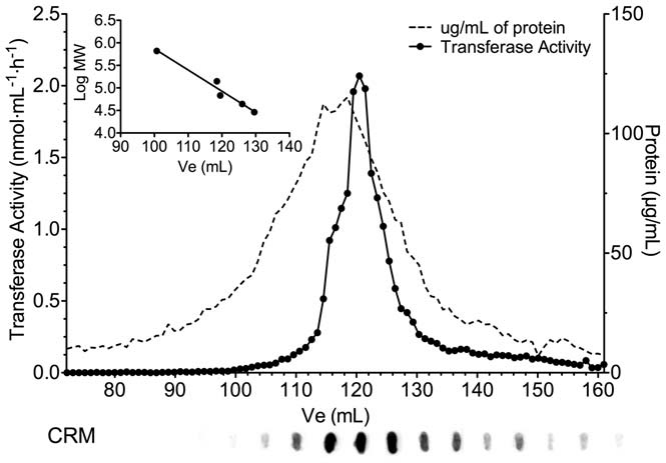
Proteins extracted from HeLa cells permanently expressing N-acetyltransferase-His8Flag were separated by molecular size exclusion chromatography on a 90×1.5 cm Sephacryl S-400 column eluted at 4°C. Each 1 mL fraction (X-axis) was analyzed for transferase activity (nmol/mL* h, left Y-axis, solid line) and total protein (µg/mL, right Y-axis, dashed line). Additionally every fifth fraction was analyzed for N-acetyltransferase protein by dot-blot using the N-terminal antibody (shown below the X-axis).

The native apparent Mr of N-acetyltransferase was determined by molecular size exclusion chromatography on a 90×1.5 cm Sephacryl S-400 column eluted at 4°C. The transferase activity eluted as a single peak with a shoulder on the high Mr side of the curve. The estimated Mr of the protein at the peak of its activity was 80 kDa (Fig. 11). Dot blots with the N-terminal antibody showed that no detectable inactive N-acetyltransferase protein was eluted in any other fractions. These data indicate that N-acetyltransferase functions as a monomer.

**Figure 12:**
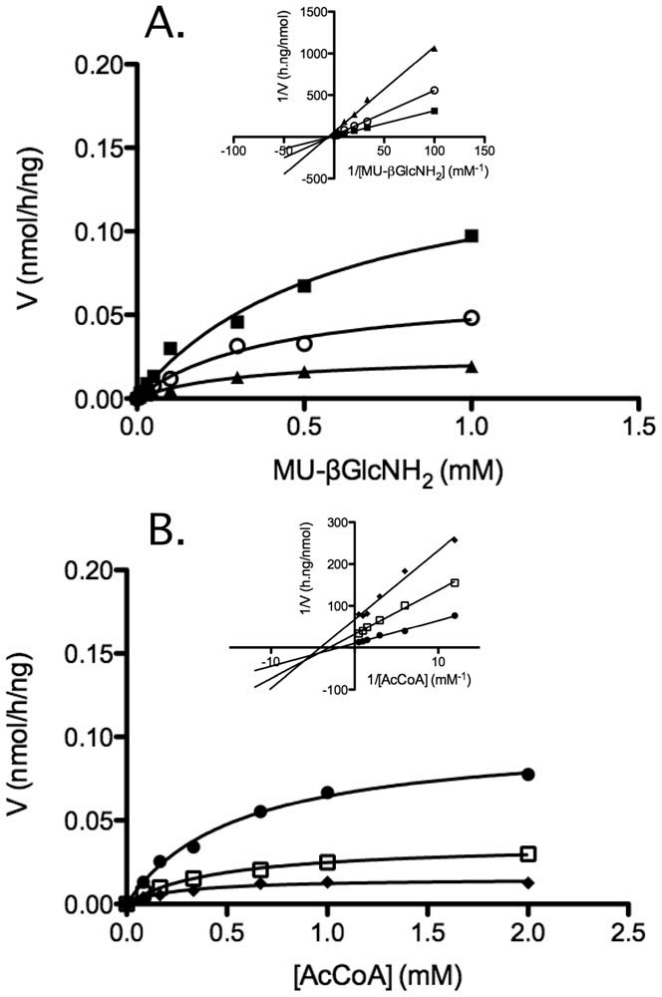
Direct Michaelis-Menten best-fit curves of specific activity (nmol/h*ng) versus [S] for the N-acetyltransferase reaction with graphic insets displaying the corresponding Lineweaver-Burk double reciprocal plots. Anti-flag affinity column purified N-acetyltransferase was used and each datum point represents the average of two or three assays. (A) The concentration of MU-GlcNH_2_ was varied between 0.01 and 1 mM, while AcCoA was fixed at 0.17 mM (▴), 0.33 mM(○) or 1.0 mM (▪). (B) The concentration of AcCoA was varied between 0.083 and 2.0 mM while MU-GlcNH_2_ was fixed at 0.05 mM (⧫), 0.10 mM (□) or 0.30 mM (•). The experiment was independently repeated three times with a representative set of graphics shown.

The kinetics of the transferase reaction were re-examined using the optimized assay procedure and the affinity purified N-acetyltransferase protein. First, three fixed amounts of either acetyl-CoA (Fig. 12A, 0.17, 0.33 or 1.0 mM) or MU-GlcNH_2_ (Fig. 12B, 0.05, 0.10 or 0.30 mM) were assayed with increasing concentrations of the other substrate and directly fitted to the Michaelis-Menten equation (Fig. 12). These data were then used to construct the double-reciprocal plots according to Lineweaver and Burk (Fig. 12A & B, insets). The resulting best-fit lines were not parallel, which would have indicated a ping-pong mechanism, but intersecting. Thus, in agreement with Meikle et al. [14], these data strongly suggest a random sequential mechanism, with the formation of a ternary complex [31], rather than the previously reported ping-pong double displacement mechanism. Similarly, the Km, Vmax and kcat of N-acetyltransferase were calculated from the Michaelis-Menten equation with each substrate fixed in turn at a saturating level, i.e., AcCoA at 2 mM or Mu-GlcNH_2_ at 1 mM, and the other substrate varied as in Figure 12 (Table 1).

**Table 1:** Kinetic parameters of the N-acetyltransferase-His8Flag.

Substrate	K_m_ (mM)	V_max_ (nmol/h/ng)	k_cat_ (S^−1^)
MU-GlcNH_2_ 1	0.45±0.09	0.54±0.06	10.38±1.14
Acetyl-CoA^2^	0.50±0.02	0.38±0.01	7.33±0.10

1 Concentrations were varied between 0.01 and 0.5 mM while the AcCoA concentration was fixed at 2 mM.

2 Concentrations were varied between 0.083 and 2 mM while the MU-GlcNH_2_ concentration was fixed at 1 mM.

Since two groups have published data supporting the formation of an acetylated-N-acetyltransferase intermediate using radiolabeled acetyl-CoA [8], [9], [12], [13], and thus a ping-pong reaction mechanism, an attempt was made to detect such a labeled intermediate utilizing N-acetyltransferase-His8Flag immobilized on the anti-Flag column. The column immobilized ∼300 nmol/h of transferase activity, or ∼1 µg of purified N-acetyltransferase-His8Flag protein, from extracts obtained from two 15 cm plates of transfected HeLa cells. Based on the specific activity of the [^3^H]acetyl-CoA that was used and a molar ratio of 1∶1 binding, ∼6,000 cpm more should have bound to the N-acetyltransferase immobilized on beads, than to control beads treated with a similar amount of untransfected HeLa cell extract. Table 2 demonstrates that there was no measurable increase in the levels of [^3^H] bound on the beads containing the immobilized N-acetyltransferase. These results are consistent with the previous conclusion made from the kinetic data (Fig. 12), i.e., that a ping-pong mechanism is not used and that no acetylated-N-acetyltransferase intermediate is formed during the enzyme reaction.

**Table 2:** Immobilized [3H]acetylated N-acetyltransferase-His8Flag intermediate cannot be detected in immunoprecipitation experiments with anti-Flag M2 beads.

With lipids^1^ at pH 6.5, 60 min	Immobilized on Flag-Beads		Unbound & washes	
	Total cpm	Activity^2^	Total cpm	Activity
Transfected^1^	1,230^3^ ±40	232 (13)	87,300±2,100	1.2
Untransfected^1^	1,240 ±60	1.1	85,000±2,100	ND^4^

Without lipids^1^ pH 6.8, 30 min	Immobilized on Flag-Beads		Unbound & washes	
	Total cpm	Activity	Total cpm	Activity
Transfected	1,150 ±150	278 (15.5)	88,000±5,500	0.6
Untransfected	1,210 ±140	0.3	92,000±4,000	ND

1 Anti-Flag beads were incubated with extracts from HeLa cell expressing (Transfected) or not expressing (Untransfected) N-acetyltransferase-His8Flag, washed and then incubated with [3H]acetyl-CoA for the given time and pH, at room temperature and with or without 1.3 mM of lipids containing 20% PI.

2 N-acetyltransferase activity in nmoles of MU produced/hour, and the concentration of protein (pmoles), calculated based on the specific activity of the purified transferase, are given.

3 The data represent three independent beads binding experiments and assays.

4 Not detectable.

## Discussion

In this report we characterize the co- and post-translational processing of N-acetyltransferase and their effects on structure and function of the enzyme. We also document the kinetic mechanism used by N-acetyltransferase, employing our optimized assay procedure, which includes negatively charged lipids in the reaction mix. Recently Durand et al. published the results of a similar study [13]. They concluded that; a) N-acetyltransferase is synthesized as an inactive precursor of 77 kDa, which contains an uncleavable signal peptide as its first TMD; b) the precursor is transported to the lysosome through conventional Tyr- and diLeu-based signals in its C-terminal cytosolic tail, with some contribution made by a second diLeu signal in a cytosolic loop near the N-terminus (Fig. 1A); c) in the lysosome the precursor is converted into a 29 kDa N-terminal alpha- and a 48 kDa ß-chain held together by disulfide bonds in the mature subunit; d) the subunits are then assembled into active ∼440 kDa oligomers; and e) acting through a ping-pong mechanism, an acetylated protein intermediate is formed from acetyl-CoA at His269 in the active site of N-acetyltransferase. Some aspects of conclusions a), c) and d) contradict conclusions that this group has made in previous publications [8], [16]. In this report we confirm and expand on conclusion c), and present strong evidence against a), d) and e). We also dispute conclusion b) based on more theoretical grounds coupled with our demonstration that the N-acetyltransferase precursor is active.

Many of the discrepancies in the numbers and apparent Mr of bands seen in Western blots of N-acetyltransferase [6], [8], [13], [32] can be explained by the tendency of multi TMD proteins to form irreversible (even with SDS) aggregates as they denature, due to heating or even an extended stay at 37°C in non-ionic detergents (Fig. 4C). This tendency can be exacerbated if the protein is extracted with certain detergents and particularly if directly extracted with 2% SDS (Fig. 3, lane3) (*e.g.*, [6]), even without heating [25]. We demonstrate that for our construct, which encodes the enzyme with a small epitope tag (Fig. 1A), bands seen on Western blots above ∼70 kDa represent irreversible aggregates and have no biological relevance (Figs. 3 & 4).

It has been shown that the apparent monomeric Mr, as determined by SDS-PAGE, of protein containing multiple TMDs, such as N-acetyltransferase, can vary as much as ±45% from that predicted from its deduced primary sequence, because of variability in the binding of SDS molecules by the TMDs [15]. Additionally the presence of oligosaccharide can increase the apparent Mr, and the N-acetyltransferase precursor chain has 5 consensus Asn-linked glycosylation sites. In the lysosome the precursor is converted to its small alpha- chain (Lys43-Asn144), with 3 glycosylation sites, and ß- chain (Gly145-Ile635), with 2 sites (Figs. 1A & 6). The data presented here are consistent with all 5 sites being occupied (Fig. 7A & B), and with the predicted 11 TMDs, which dictate the orientation of the joining loop structures, *i.e.*, with loops 1, 7 and 11 (carrying the glycosylation sites) residing in the lumen of the ER (Fig. 1A). We demonstrate that deglycosylation of the precursor chain (containing both complex and high mannose oligosaccharides) decreases its apparent Mr from 62 to 50 kDa (predicted Mr, 73 kDa with or 70 kDa without the signal peptide), the C-terminal mature ß-chain (containing all 11 TMD and only complex oligosaccharides) from 44 to 38 kDa (predicted Mr, 57 kDa) (Fig. 7B) and the alpha-chain (containing no TMD and both complex and high mannose oligosaccharides) from 27 to 12 kDa (predicted Mr, 16 kDa with and 12 kDa without the signal peptide) (Fig. 7A).

Using the N-terminal antibody and extracts from non-transfected human fibroblasts, we also demonstrated that the conversion of the single 62 kDa precursor into smaller alpha- and ß-chains is not an artifact of over expressing an epitope tag N-acetyltransferase protein (Fig. 5). Interestingly, there was no significant difference in the ratio of the precursor to the processed forms of the protein between normal fibroblasts and cells from a patient with I-cell disease. Nor was there any significant difference in the specific activity of the enzyme between these cell lines. Since I-cells fibroblasts are deficient in multiple soluble proteases [28], that depend on a mannose-6-phosphate tag [27] for transport to the lysosome (e.g., Cathepsin B, D, F, H, K, L, O, S and Z [26]), processing must occur through an as yet unidentified protease(s). Additionally these data confirm that N-acetyltransferase is not dependent on the mannose-6-phosphate receptor for transport to the lysosome (Fig. 5).

Some of the variations in apparent Mr reported here and those previously published [13] can be explained by the large C-terminal epitope tag, 7.8 kDa, used by Durand et al., compared to our 2.1 kDa His8-Flag tag. This explains the 4 kDa difference in the apparent Mr of the glycosylated ß-chain, containing the epitope tags, and the smaller 2 kDa difference in the Mr we report for the glycosylated alpha-chain, lacking the tag, but does not explain the 15 kDa difference observed in the Mr of the precursor chain (discussed below).

The amino-terminal sequence of the alpha-chain coupled with the observations that, a) it appears on Western blots as a single band, i.e., there is no larger form that could potentially include the signal peptide with a blocked N-terminus and b) its deglycosylated Mr is identical to that predicted for a peptide composed of Lys43-Asn144 (Fig. 1A), indicate that it does not contain the signal peptide and thus has no TMD. Therefore, either the signal peptide is cleaved co-translationally in the ER or is removed in the lysosome prior to the proteolytic event that produces the alpha- and ß- chains (Fig. 6). To try and differentiate between these two possibilities we attached a ten amino acid myc-tag to the amino-terminus of the signal peptide. The tag did not interfere with translocation of the nascent peptide into the ER, as judged by the presence of high mannose oligosaccharides on the protein (Fig. 8B). However, the tagged protein was unable to exit the ER for processing in the lysosome into the alpha- and ß- chains (Figs. 8A & 9C). Similarly, it has been reported that the addition of an N-terminal GFP-tag to the ordinarily cleavable signal peptide of glucagon-like peptide-1 receptor, prevented signal peptide cleavage and transport of the receptor out of the ER to the plasma membrane [33]. These data suggest that for some proteins, an N-terminal extension of their signal peptides interferes with signal peptide cleavage, which in turn prevents the proteins from exiting the ER. Significantly the ER localized precursor polypeptide containing the N-terminal myc extension expressed near normal levels of N-acetyltransferase activity (Fig. 8A), which could be immobilized on Protein-G beads containing a myc IgG. These data clearly demonstrate that the precursor is active and does not, as previously reported [13], need to be proteolytically processed and assembled into oligomers or multienzyme complexes in the lysosome to become functional.

Many lysosomal proteins undergo posttranslational proteolytic processing in the late endosome/lysosome, which can include both endo- and exo-proteolytic fragmentation. However, in only a few cases does the fragmentation process involve the activation of a zymogen. Usually such cases involve a lysosomal protease, e.g. cathesin D, and likely represent a cellular protective mechanism. On the other hand, many glycosidases undergo multiple proteolytic processing events that lead to no observable biochemical changes in their function (reviewed in [34]), e.g. the alpha- and ß-subunits of Hex A [30]. The proteolytic processing of the Hex A subunits produce several active intermediate forms with different pIs, originally thought to be unique Hex isozymes. These event have been characterized by pulse chase experiments in human fibroblasts [35], [36] and by Edman degradation of isolated “intermediate isozyme forms” from human placenta [37]. These events represent the digestion of surface loop structures once they have been exposed to the lysosomal environment [38], [39]. Such loops were likely only needed during protein folding and disulfide bond formation in the ER.

In order to differentiate between the co- or post-translational removal of the signal peptide, an N-terminal construct containing the signal peptide and most of the N-terminal luminal loop structure (Fig. 1B) was expressed in HeLa cells. The expressed protein was soluble, but remained in the ER (Fig. 9D & E). The protein was also fully deglycosylated by endo-H digestion (Fig. 10), resulting in an apparent Mr nearly identical to that predicted for the protein devoid of the 2.6 kDa signal peptide, i.e., 17 kDa (Figs. 1B & 10). From these and data from the N-terminal myc construct, we conclude that the signal peptide is cleaved co-translationally and that this event is necessary for transport of full length N-acetyltransferase out of the ER, but not for folding into a functional precursor enzyme.

There are two possible translational start sites predicted from the genomic sequence of N-acetyltransferase, one encodes a 58 residue “long” signal peptide (Fig. 1A, SP-L) and the other a 30 residue “short” peptide (Fig. 1A, SP-S) [13]. The short sequence is the only one found in a spliced EST data base and was thus used by ourselves [4] and other [6] as the initiating ATG in our N-acetyltransferase cDNA expression constructs (the amino acid numbering used here assumes this ATG encodes Met1). The group represented by Feldhammer et al. [16] and Durand et al. [13] primarily used the long sequence, containing both ATGs, to express N-acetyltransferase and conclude that the long signal peptide is retained in the enzyme. However, our data clearly demonstrates that the mature alpha-chain does not contain the short signal peptide. Thus, assuming that the long signal peptide was not removed co-translationally in the ER we would expect to see its removal during the pulse-chase experiments reported by Durand et al. [13]. Depending on whether the initial cleavage occurred at Lys43 or Gly145, a decrease of 6.7 kDa (the Mr of the sequence between the first ATG of the long signal peptide and Lys43 (Fig. 1A)) in either the precursor or the mature alpha-chain should have been observed. However, their data failed to show such a decrease during the chase period in either protein band [13]. While the apparent Mr they report for the mature alpha-chain is only 2 kDa larger than the 27 kDa we report, their precursor chain is ∼15 kDa heavier (77 versus 62 kDa). Whereas the 2 kDa difference is insignificant, the ∼15 kDa is not, even taking into account their ∼6 kDa larger C-terminal tag. However, if the signal peptide was to be removed when protein initiation occurs at the second ATG (short), allowing transport to the lysosome for processing, but was retained if the first ATG (long) was used, preventing transport out of ER (similar to the effects we observe after adding the myc tag to the short signal peptide), this would increase the Mr by an additional 5.4 kDa, which would result in an insignificant ∼4 kDa difference. For this explanation to be correct protein initiation would have to occur at both the first and second ATGs in their expression construct. This is theoretically possible as the group synthesized the extension and part of the short signal peptide in order to make their expression construct and reportedly included a HindIII restriction site directly upstream of the first initiating ATG, *i.e.*, AAG**C**TTATGA in their long signal peptide. They also convert the sequence downstream of the second initiating ATG site from the wild-type **G**GCATGA, which is used in this work, to **G**GCATGT [5]. Importantly, the addition of the HindIII site results in the generation of a very poor Kozak consensus sequence at the first ATG site. The optimum sequence for initiation by eukaryotic ribosomes is **A**CCATGG with a purine (A/G) in the dominant **-3** position. If the first ATG is present in a weak Kozac sequence it may be skipped and translation can begin at a down stream ATG with a stronger sequence [40]. When a pyrimidine replaces the purine in the -3 position, translation also becomes more sensitive to changes in positions -1, -2, and +4 [40]. Previously reported experimental data also support this hypothesis. Feldhammer et al. reported that their 77 kDa precursor band was reduced to 67 kDa when treated with either endo-H or PNGase, indicating that it contained only high mannose oligosaccharides, consistent with the majority of the protein being localized in the ER. However, consistent with our data for the mature ß-chain (Fig. 6B), they also reported the presence of a weak 48 kDa band containing only complex oligosaccharides [16]. The 62 kDa N-acetyltransferase precursor protein we expressed with the short signal peptide contained both high mannose and complex carbohydrates (Fig. 7A & B) and was found primarily in lysosomes (Figs. 6 & 9A). An N-acetyltransferase precursor protein containing fully endo-H sensitive oligosaccharides was only seen when the myc N-terminal tag was added (Fig. 8B). This protein was not transported to the lysosome (Fig. 9B), but retained in the ER (Fig. 9C). Furthermore, no myc-alpha-chain was detected with the myc-antibody (Fig. 8B), nor was any mature alpha-chain detected with the N-terminal antibody (Fig. 8A). During the generation of the myc-N-acetyltransferase construct, the sequence surrounding our original ATG, initiating the short signal peptide, was replaced by **C**TGATGA (a weak Kozac sequence) and the new initiating site for the myc extension was **G**CCATGG, consistent with translation primarily occurring at this upstream site [40].

A putative YXXØ lysosomal targeting motif, where Ø is a large hydrophobic residue and X is any amino acid, was identified by Durand et al., LIAYILY627 (Fig. 1A), as residing in the C-terminal tail of N-acetyltransferase [13]. However, for several reasons we believe that this putative signal sequence is unlikely to be functional. Firstly, this sequence motif has a statistical probability of appearing in many proteins, but for it to be functional it must also be accessible for interacting with the components of the cell's sorting machinery, *i.e.*, it must not be folded into the structure of the protein [41]. However, this putative N-acetyltransferase transport signal is predicted by Durand et al. [13], Feldhammer et al. [16] and ourselves [4] (using the TMHMM Server v.2.0) to comprise the C-terminal section of the final TMD (residues 608–627) of N-acetyltransferase (Fig. 1A). For lysosomal-sorting, these types of signal are located 6–9 residues from the final TMD [41]. Secondly, although X-X can be “any amino acids”, hydrophilic amino acids are favored and for lysosomal-sorting, acidic residues are generally found in the X positions, as is an important Gly preceding the critical Tyr (Y) residue. Thirdly, another Tyr residue has not been reported in the Ø position of any other functional targeting sequence [41]. Fourthly, Durand et al. mutated their putative critical Tyr (Y624A) and the protein was still processed, which should have eliminated this as a targeting motif [41]. It was only when the entire C-terminal sequence was deleted (del 624–635) that N-acetyltransferase failed to be processed or produced any transferase activity. This large deletion is predicted by the TMHMM Server to destabilize the final TMD, resulting in the C-terminus of N-acetyltransferase-del 624–635 residing in the lumen of the ER, i.e., the protein is likely misfolded. Finally, an important control for the use of *in vitro* mutagenesis as a means to identify transport signals is that the resulting protein retains its functionality. Since Durand et al. mistakenly identified the N-acetyltransferase precursor as inactive, this important control was not considered. Indeed re-evaluation of their data suggests the true signal for intracellular transport is likely the ETDRLI209 sequence that they identified in the first large cytosolic loop of N-acetyltransferase (Fig. 1A). This fits the majority of criteria for one of the two classes of di-Leu repeat signals, [DE]XXXL[LI], which does not need to be present in the C-terminal tail in order to function [41]. Durand et al. reported that mutations of the critical Leu and/or Ile resulted in little N-acetyltransferase processing and near normal transferase activity [13].

Although we agree that the N-acetyltransferase precursor is cleaved in the lysosome into mature alpha- and ß- chains, Durand et al. estimated that the cleavage occurred within the end of the first and/or beginning of the second lysosomal loop, based on tryptic fragments identified by mass spectrometry [13]. The amino-terminal sequencing reported here directly places the mature amino terminus of both the alpha- (Lys43) and ß- (Gly145) chains within the first intra-lysosomal loop of N-acetyltransferase (Fig. 1A). Additionally, the weak cross-reactivity of the ß-chain with the N-terminal antibody (Figs. 4, 5, 6, 7), made against residues 53–156, is consistent with cleavage in the first lysosomal loop not the second (Fig. 1A).

We examined the native Mr of N-acetyltransferase by conventional standing molecular sieve chromatography with Sephacryl S-400. To prevent aggregation the separation was carried out at 4°C and the elution buffer contained 0.02% DDM. The resulting calculated Mr of 80 kDa indicates that N-acetyltransferase functions as a monomer (Fig. 11). Additionally since N-acetyltransferase, purified on a Flag antibody column, is fully functional (in the presence of negatively charged lipids, Fig. 2) and protein stained SDS-PAGE gels show only band that are also detected by either the Flag or the N-terminal N-acetyltransferase antibodies (Fig. 4), we conclude that the protein encoded by the *HGSNAT* gene is both necessary and sufficient to produce active N-acetyltransferase *in vivo*, as well as *in vitro*.

Finally we re-examined the kinetic mechanism used by N-acetyltransferase and found, in agreement with Meikle et al. [14], that it was not a ping-pong double displacement, but a random-order ternary-complex mechanism (Fig. 12). These data were confirmed by demonstrating that no [^3^H]acetylated-N-acetyltransferase intermediate was formed using ∼1 µg of fully functional, purified and immobilized N-acetyltransferase (Table 2). Thus, although we cannot rule out the possibility that His269 is part of the active site of N-acetyltransferase, our data clearly demonstrate that its acetylation, as determined by mass spectrometry [13], is not likely to be biologically relevant.
